# Predicting protein-nucleic acid interactions via protein language models with biophysical and evolutionary priors

**DOI:** 10.1016/j.isci.2026.115795

**Published:** 2026-04-17

**Authors:** Zidong Su, Xiaochun Zhang, Boxue Tian

**Affiliations:** 1 MOE Key Laboratory of Bioinformatics, State Key Laboratory of Molecular Oncology, Beijing Frontier Research Center for Biological Structure, School of Pharmaceutical Sciences, Tsinghua University, Beijing 100084, China

**Keywords:** biophysics, computational bioinformatics, machine learning

## Abstract

Protein interactions with nucleic acids are fundamental to numerous biological processes. Here, we present PNABPred, a multi-modal framework that integrates biophysical and evolutionary priors into a protein language model to predict protein-nucleic acid interactions. Specifically, semantic representations are combined with classical evolutionary representations and biophysical representations. Our results showed that PNABPred outperforms other state-of-the-art sequence-based methods. In RNA-binding protein classification tasks, PNABPred achieved an Matthews correlation coefficient (MCC) of 0.889 and area under the receiver operating characteristic curve (AUROC) of 0.990, 17.44% and 3.23% higher than the next best method (Seq-RBPPred), respectively. In DNA-binding site prediction, PNABPred also outperformed the transformer-based method CLAPE-DB by 20.31% and 4.65% (MCC 0.468; AUROC 0.922) on the Test_129 dataset. PNABPred employs only protein sequences as inputs, identifying nucleic acid binding sites even in intrinsically disordered regions. This framework supports scalable sequence screening and annotation of nucleic acid-binding proteins for basic research, biotechnology, and therapeutic development applications.

## Highlights

• PNABPred is a multi-modal framework predicting protein-nucleic acid interactions
• Biophysical and evolutionary priors enhance protein language models for nucleic acid binding
• PNABPred outperforms current sequence-based methods in predicting binding sites
• Using only protein sequences, PNABPred identifies binding sites in disordered regions

## Graphical abstract

**Figure:**
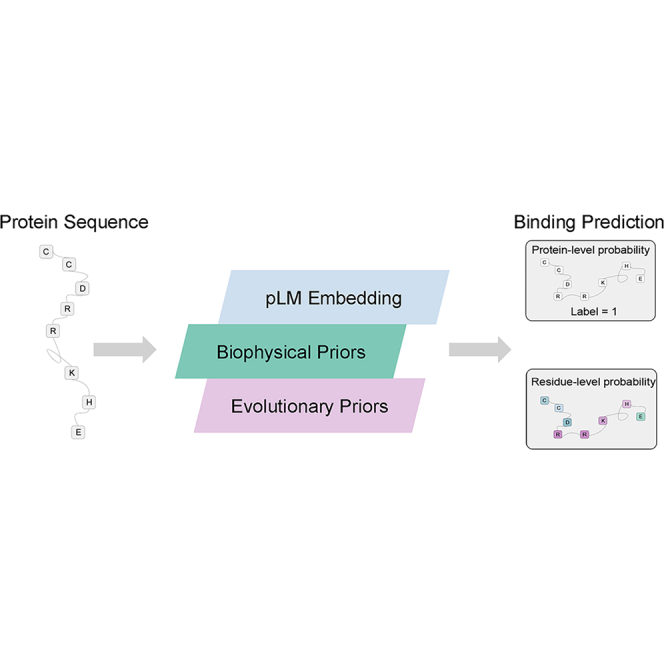

## Introduction

DNA- or RNA-binding nucleic acid-binding proteins (NABPs) have been ubiquitously identified in all domains of life and mediate essential biological processes such as transcription, replication, signal transduction, RNA processing, and defense against pathogens, among others.^1^^,^^2^^,^^3^ Therefore, accurately predicting whether proteins bind to nucleic acids and identifying their binding sites is an essential step in both fundamental biology and drug development research.^4^^,^^5^ Despite a wealth of experimental techniques for testing and validating protein structural and functional properties, these approaches^6^^,^^7^^,^^8^^,^^9^ are labor-intensive and benefit considerably from hypothesis-forming computational approaches to predicting protein-nucleic acid interactions.

Numerous Machine Learning Models (MLMs) for classifying NABPs and identifying their respective binding sites have been established,^10^^,^^11^^,^^12^ employing features derived from amino acid composition or physicochemical properties.^13^^,^^14^ Additionally, incorporating evolution-inspired priors has been demonstrated to substantially enhance prediction of protein-nucleic acid binding sites.^15^ More recently, deep learning techniques such as DeepDISE^16^ employ convolutional neural networks (CNNs) to capture local sequence patterns or use recurrent neural networks (RNNs) to model temporal dependencies, as in EL_ long short-term memory (LSTM),^17^ apply multi-layer perceptrons (MLPs) for NABP classification, for example NucleicNet,^18^ or integrate sequence and structural features to enhance performance with graph neural networks (GNNs), such as GraphBind.^19^ Alternatively, protein language models (pLMs), such as ESM-2^20^ and ProtTrans,^21^ show stronger classification accuracy in protein structural, functional, and evolutionary prediction tasks by capturing features concealed within sequence data through large-scale unsupervised learning. In our previous study, we developed the CLAPE^22^ method using ProtTrans, while Zhu et al. predicted DNA-binding sites using an LSTM-attention network embedded with three pLM,^23^ and Zeng et al. retrained ESM-2 with UniDBP.^24^

Despite these advances, contemporary models, including those leveraging pLMs, still exhibit limitations, including reduced generalizability for tasks involving high sequence diversity and the ability to recognize atypical NABPs.^25^ A logical approach to augment sequence-based methods is to incorporate structural information.^26^ However, while tools like AlphaFold^27^^,^^28^^,^^29^ have achieved breakthroughs in single-chain protein structure prediction, NABP models using AlphaFold2-predicted structures as input (e.g., GraphBind and EquiPNAS^30^) show reduced prediction accuracy compared to ground-truth structural data and struggle to handle protein regions lacking a stable tertiary structure, for example intrinsically disordered regions (IDRs). Thus, improving the predictive performance of models relying solely on protein sequence remains a potentially fruitful research objective. However, these overall gaps in the predictive capacity of existing sequence-based methods imply that their feature representations, while sophisticated, may not yet successfully integrate diverse features from biophysical properties and evolution-inspired priors that may complement those features learned by pLM.^31^ Therefore, the primary objective of this work is to develop a framework that integrates these diverse biophysical and evolutionary features to enrich the model’s representations, thereby improving the accuracy and generalizability of NABP prediction.

Here, we present PNABPred, a multi-modal framework that integrates biophysical and evolution-inspired priors into a pLM to predict protein-nucleic acid interactions. PNABPred predicts protein-side binding residues, not the specific nucleic acid sequence (e.g., motifs) that the protein recognizes. Specifically, semantic representations are combined with classical evolutionary representations and biophysical representations. In extensive benchmarking against published datasets, the PNABPred framework demonstrated higher accuracy than other current NABP models in predicting nucleic acid binding capacity and identifying specific binding sites for both DNA and RNA substrates. These results verify the superior accuracy and generalizability of our multimodal feature strategy for NABP modeling compared to other methods. The PNABPred framework thus offers a powerful tool for computational analyses of protein-nucleic acid interactions.

## Results

### PNABPred framework

**Figure 1:**
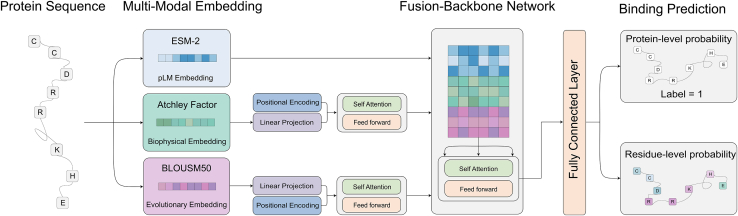
Schematic of the PNABPred framework The model processes a protein sequence via a multi-modal embedding module to generate three complementary representations: pLM embeddings from ESM-2, biophysical embeddings from Atchley factors, and evolution-inspired priors embeddings from BLOSUM50. Both biophysical and evolution-inspired prior embeddings undergo initial linear projection and addition of positional encoding to preserve sequence order, followed by intra-modal refinement. These features are concatenated and fed into a transformer-based backbone network that utilizes a self-attention mechanism to integrate the multi-modal features. Finally, classification heads predict protein-level (label of 1 indicates a binding protein, while label of 0 indicates a non-binding protein) and residue-level binding probabilities.

The PNABPred design was aimed at accommodating two distinct prediction tasks fundamental to protein-nucleic acid interaction research, including protein-level classification as an NABP or non-NABP (i.e., whether or not a protein can bind nucleic acids), and residue-level prediction of specific amino acids involved in nucleic acid binding. The framework is based on a multi-modal feature fusion strategy that combines a pLM with biophysical and evolutionary features of proteins. The framework consists of two primary modules: a feature embedding module that generates three complementary representations of the sequence and a transformer-based backbone network that integrates these features to make a final prediction (Figure 1). Specifically, PNABPred predicts protein residues involved in nucleic acid binding but does not infer the sequence preferences of the nucleic acid substrate. First, PNABPred leverages the pre-trained ESM-2 pLM to generate embeddings that capture features within protein sequences that are relevant to nucleic acid interactions. PNABPred also incorporates Atchley factors^32^ derived from biophysical amino acid properties (such as polarity, secondary structure propensity, molecular volume, codon diversity, and electrostatic charge) that contribute to governing molecular interactions. Finally, the framework incorporates evolution-inspired priors through embeddings derived from the BLOSUM50 substitution matrix,^33^ which provides context-independent log-odds scores for amino acid substitutions observed in conserved protein blocks. Features obtained through this module should capture long-term evolutionary constraints and tolerated mutations, which are subsequently fused to features from other modules and processed by the transformer-based backbone module. The self-attention^34^ mechanism can model complex, long-range dependencies between residues and effectively integrate disparate signals among the pLM, biophysical, and evolutionary features. Extending beyond simple feature concatenation, this design allows the model to learn relationships between local biophysical characteristics, evolutionary context, and global representations of the protein to enhance NABP prediction accuracy.

### PNABPred improves the accuracy of protein-nucleic acid binding prediction

**Figure 2:**
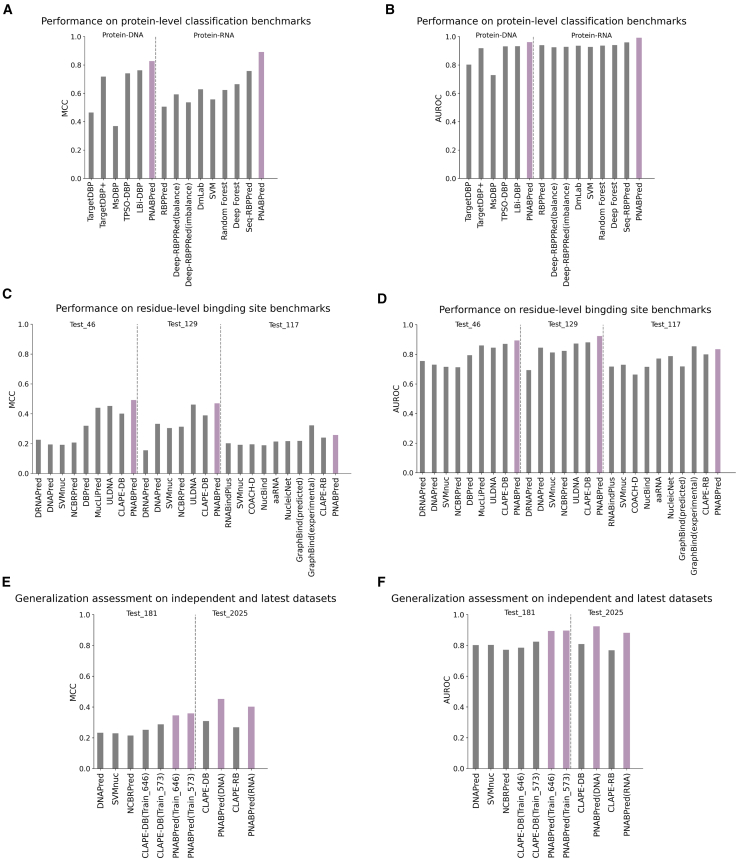
PNABPred achieves better performance and generalization (A and B) Performance in classifying proteins as DNA-binding or RNA-binding on benchmark datasets, measured by MCC and AUROC, respectively. The model was evaluated on the UniSwiss dataset for DNA-binding proteins (left) and the RBPPred/Seq-RBPPred datasets for RNA-binding proteins (right) (C and D) Performance in identifying specific DNA- and RNA-binding residues on benchmark datasets, measured by MCC and AUROC. Evaluations were conducted on standard benchmarks: Test_46 and Test_129 for DNA-binding sites and Test_117 for RNA-binding sites. (E and F) Rigorous generalization assessment. The analysis includes the independent Test_181 dataset (left), where PNABPred and CLAPE-DB were cross-trained on different source sets (Train_646 vs. Train_573) and a newly-curated dataset from BioLiP (right), both measured by MCC and AUROC.

To evaluate the accuracy of PNABPred, we first conducted protein-level classification tasks to predict whether a protein could bind nucleic acid on two benchmark datasets comprised of DNA-binding proteins (DBPs) or RNA-binding proteins (RBPs), respectively (Figure 2; Table 1). On the DBP dataset, the Matthews correlation coefficient (MCC) and area under the receiver operating characteristic curve (AUROC) of PNABPred were 0.825 and 0.959, representing improvements of 8.27% and 2.90% over LBi-DBP and 11.34% and 3.01% over TPSO-DBP, both of which are based on bidirectional long short-term memory (BiLSTM) architectures. On the RBP benchmark dataset, PNABPred’s MCC and AUROC were 0.889 and 0.990, respectively, surpassing the RNN model Seq-RBPPred by 17.44% and 3.23% and the CNN model Deep-RBPPred (balance) by 50.17% and 7.03%. The results suggest that our multi-modal feature integration strategy enables PNABPred to achieve high prediction accuracy.

**Table 1:** Test performance on protein-level classification benchmarks

	Models	MCC	AUROC
Protein–DNA	TargetDBP	0.465	0.802
	TargetDBP+	0.718	0.918
	MsDBP	0.369	0.728
	TPSO-DBP	0.741	0.931
	LBi-DBP	0.762	0.932
	PNABPred	**0.825 ± 0.0014**	**0.959 ± 0.0011**
Protein–RNA	RBPPred	0.506	0.939
	Deep-RBPPRed (balance)	0.592	0.925
	Deep-RBPPRed (imbalance)	0.536	0.928
	DmLab	0.628	0.935
	SVM	0.557	0.927
	Random Forest	0.623	0.936
	Deep Forest	0.664	0.940
	Seq-RBPPred	0.757	0.959
	PNABPred	**0.889 ± 0.0019**	**0.990 ± 0.0007**

Bold entries indicate the highest prediction accuracy on the test dataset.

Values are taken from ref.^35^^,^^36^^,^^37^^,^^38^^,^^39^^,^^40^^,^^41^^,^^42^

Next, we evaluated PNABPred’s performance in predicting protein-nucleic acid binding sites across benchmark datasets (Figure 2; Table 2). For DNA binding site prediction, PNABPred consistently outperformed existing sequence-based models across two standard benchmarks, Test_46 and Test_129. On Test_46, PNABPred achieved an MCC of 0.490 and AUROC of 0.892, which were 22.19% and 2.41% improvements over the pLM-based method CLAPE-DB and 8.41% and 5.56% over ULDNA, respectively. On the Test_129 dataset, PNABPred outperformed CLAPE-DB by 20.31% and 4.65% and surpassed ULDNA by 1.52% and 5.61% with an MCC of 0.468 and AUROC of 0.922. Notably, PNABPred showed even greater improvement over older methods, with 136.71% and 49.52% higher MCC and 25.11% and 12.13% higher AUROC than NCBRPred on these benchmark datasets. For RNA binding site prediction on the Test_117 dataset, PNABPred achieved MCC and AUROC values of 0.256 and 0.833, respectively, exceeding CLAPE-RB by 6.67% and 4.13%.

**Table 2:** Test performance on residue-level binding prediction benchmarks

	Datasets	Models	Pre	F1	MCC	AUROC
Protein–DNA	Test_46	DRNAPred	0.185	0.291	0.226	0.755
		DNAPred	0.157	0.254	0.194	0.730
		SVMnuc	0.154	0.250	0.192	0.715
		NCBRPred	0.165	0.265	0.207	0.713
		DBPred	0.243	0.362	0.320	0.794
		MucLiPred	0.202	0.317	0.440	0.860
		ULDNA	0.435	0.502	0.452	0.845
		CLAPE-DB	0.306	0.434	0.401	0.871
		PNABPred	**0.545 ± 0.010**	**0.534 ± 0.015**	**0.490 ± 0.016**	**0.892 ± 0.0001**
	Test_129	DRNAPred	0.190	0.210	0.155	0.693
		DNAPred	0.353	0.373	0.332	0.845
		SVMnuc	0.371	0.341	0.304	0.812
		NCBRPred	0.392	0.347	0.313	0.823
		ULDNA	0.491	0.496	0.461	0.873
		CLAPE-DB	0.396	0.427	0.389	0.881
		PNABPred	**0.501 ± 0.003**	**0.498 ± 0.001**	**0.468 ± 0.001**	**0.922 ± 0.0001**
Protein–RNA	Test_117	RNABindPlus	0.227	0.248	0.202	0.717
		SVMnuc	0.240	0.235	0.192	0.729
		COACH-D	0.252	0.235	0.195	0.663
		NucBind	0.235	0.233	0.189	0.715
		aaRNA	0.166	0.237	0.214	0.771
		NucleicNet	0.201	0.261	0.216	0.788
		GraphBind (predicted structures)	0.303	0.171	0.218	0.718
		GraphBind (experimental structures)	0.294	**0.358**	**0.322**	**0.854**
		CLAPE-RB	0.201	0.281	0.240	0.800
		PNABPred	**0.318 ± 0.007**	**0.293 ± 0.001**	**0.256 ± 0.001**	**0.833 ± 0.001**

Bold entries indicate the highest prediction accuracy on the test dataset.

Values are taken from ref.^15^^,^^18^^,^^19^^,^^22^^,^^23^^,^^43^^,^^44^^,^^45^^,^^46^^,^^47^^,^^48^^,^^49^

Additionally, we compared our sequence-based model with GraphBind, which utilizes 3D structural information. On Test_117, GraphBind achieved higher accuracy when using experimentally determined structures. However, because experimental structures are often unavailable, PNABPred outperformed GraphBind when it used AlphaFold2-predicted structures, achieving 17.43% and 16.02% higher MCC and AUROC, respectively. Across all evaluations, PNABPred showed significantly higher accuracy than other models (*p* < 0.05, Mann-Whitney U test). These results suggest that PNABPred can capture binding features in primary protein sequence relevant to nucleic acid binding more effectively than a geometric model relying on imperfect or noisy predicted structures.

### PNABPred has better generalization ability

To rigorously assess the real-world applicability and robustness of PNABPred, we tested its ability to generalize to recent and diverse protein data and its stability across different training conditions. First, we constructed two new, non-redundant test sets using protein-nucleic acid complexes deposited in the BioLiP^50^ database up to January 2025, ensuring a 30% sequence identity cutoff (Table S3). On this new DNA binding site dataset, PNABPred achieved an MCC of 0.451 and AUROC of 0.922, outperforming CLAPE-DB by 45.95% and 14.11%, respectively. Similarly, tests on the corresponding RNA binding site dataset yielded an MCC of 0.400 and AUROC of 0.880, surpassing CLAPE-RB by 49.25% and 14.58% (Figure 2; Table S4). This strong performance on contemporary data thus demonstrated that PNABPred could learn generalizable features of nucleic acid binding, rather than just identifying patterns in the benchmark datasets.

To further probe its generalizability, we conducted cross-training experiments in which PNABPred and CLAPE-DB were each trained on the Train_646 or Train_573 standard training sets, then evaluated, along with other models, on Test_181^51^ (Figure 2; Table S4). After training with Train_646, PNABPred achieved an MCC of 0.344 and an AUROC of 0.892, 36.51% and 8.31% higher than CLAPE-DB trained on the same dataset. Similarly, PNABPred trained with Train_573 showed an MCC of 0.357 and an AUROC of 0.894, 24.39% and 13.89% improvements over the CLAPE-DB counterpart. These consistently and significantly higher scores indicated that PNABPred demonstrates a robust and reliable capacity for predicting nucleic acid binding, regardless of the specific training set composition and data distribution.

### Analysis of module contribution and model feature representation

**Figure 3:**
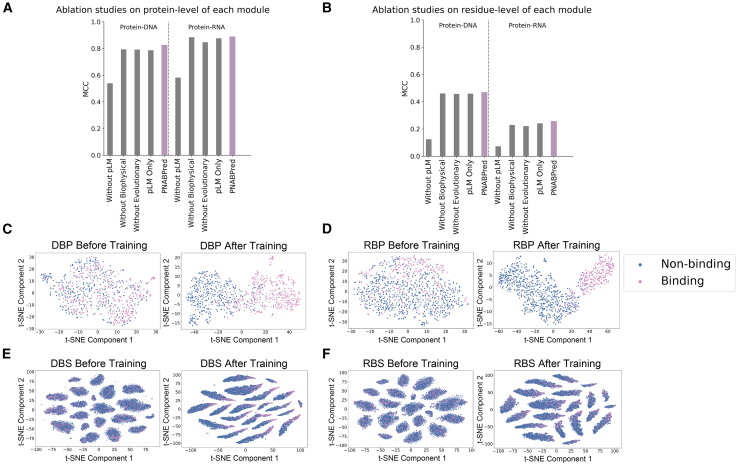
Ablation studies and feature space visualization reveal the contribution of each module to PNABPred’s performance (A and B) Ablation studies quantifying the contribution of each feature module. The charts show the drop in MCC performance for protein-level classification (A) and residue-level prediction (B) when specifically removing the protein language model (pLM), biophysical features (Atchley factors), or evolution-inspired priors (BLOSUM50). (C–F) t-SNE visualization of the feature space before and after training. (C, D) Feature distributions for protein-level classification (DBP and RBP). (E, F) Feature distributions for residue-level binding site prediction (DBS and RBS). Blue dots represent non-binding samples (negative), and pink dots represent binding samples (positive).

To assess the quality of its feature representations and the contribution of each module to prediction accuracy, we conducted ablation studies of PNABPred (Figure 3; Tables S7 and S8) by systematically removing the pLM (ESM-2), biophysical (Atchley Factors), or evolutionary (BLOSUM50) modules, as well as evaluating a model using only pLM features. Measuring the impact of each module on accuracy in protein-level classification on the above DBP and RBP benchmarking sets, we found that the full model achieved an MCC of 0.825 for DBPs and 0.889 for RBPs. Upon removing the pLM module, MCC decreased to 0.539 for DBP classification and 0.582 for RBP classification. The model relying only on pLM features achieved an MCC of 0.785 (DBP) and 0.876 (RBP). Without biophysical features, MCCs dropped to 0.793 (DBP) and 0.884 (RBP). Similarly, without evolution-inspired priors, MCCs declined to 0.792 (DBP) and 0.847 (RBP). This decrease in accuracy was also reflected in residue-level binding site prediction tasks. In DNA binding site predictions on Test_129 and RNA binding site predictions on the Test_117 dataset, the full model achieved MCCs of 0.468 (DBS) and 0.256 (RBS). Removal of the pLM module resulted in decreasing MCC scores to 0.124 (DBS) and 0.072 (RBS). The pLM-only model yielded MCCs of 0.459 (DBS) and 0.241 (RBS). These results confirm that ESM-2 embeddings form the basis of PNABPred predictions. However, eliminating the biophysical or evolutionary features also led to significantly lower performance. In DNA binding site predictions on Test_129, in the absence of biophysical features, MCC decreased from 0.468 to 0.460 and similarly fell to 0.457 following removal of evolutionary features. In RNA binding site prediction on Test_117, removing biophysical or evolutionary features decreased the MCC from 0.256 to 0.239 and 0.221, respectively. Although less severe than removal of the pLM module, these decreases represent a substantial loss of predictive capability, thus confirming that each module substantially contributes to improving the accuracy of PNABPred.

Additionally, we evaluated alternative backbone architectures for comparison with the transformer model (Figure S1; Table S9). In parallel trials with an RNN and a simple MLP, PNABPred consistently outperformed the other models across all metrics. Although RNNs are designed to handle sequential data, both protein-level and residue-level binding prediction tasks rely heavily on capturing global context and long-range dependencies. The transformer’s self-attention mechanism weighs the influence of every residue on every other residue, regardless of their distance in the primary sequence, thus providing a uniquely adept tool for identifying context and dependencies in protein sequences. These results suggest that a transformer-based architecture is therefore an effective approach for fusing multi-modal features and modeling complex relationships in protein recognition of nucleic acid sequences.

To qualitatively assess the representations learned by PNABPred, we used t-distributed stochastic neighbor embedding^52^ (t-SNE) to visualize the feature space (Figure 3). For both the protein-level classification and residue-level binding site prediction tasks, the features learned by the trained PNABPred model formed distinct, well-separated clusters corresponding to different classes or nucleic acid binding residues. In contrast, prior to training, the features exhibited a random distribution, lacking identifiable clusters. Visualization of the original features generated by ESM-2 (Figure S2) showed no obvious separation among categories prior to model processing. This visualization indicates that the PNABPred architecture learns to organize the initial features into a new embedding space where binding and non-binding entities form distinct, separable clusters. Statistical analysis of the benchmark data confirmed that proteins in these datasets shared the above-reported biases in amino acid composition at interaction interfaces. Further analysis of amino acid distributions at binding sites predicted by PNABPred revealed (Figure S3) that the model correctly identified strong enrichment for R, K, and W at predicted DNA binding sites and for R, K, and H at predicted RNA binding sites, thus showing obvious consistency with known principles of protein-nucleic acid interactions. To quantify the degree of consistency between our model and ground truth, we calculated Kullback-Leibler (KL) divergence between the amino acid distributions at true binding sites and binding sites predicted by the model. The KL divergence was near zero for both DNA (forward: 0.0185, reverse: 0.0168) and RNA (forward: 0.0217, reverse: 0.0202) sites. These results confirm that PNABPred can capture statistical correlations relevant to biophysical properties required for molecular recognition.

### Case studies illustrate PNABPred’s accuracy on diverse proteins

**Figure 4:**
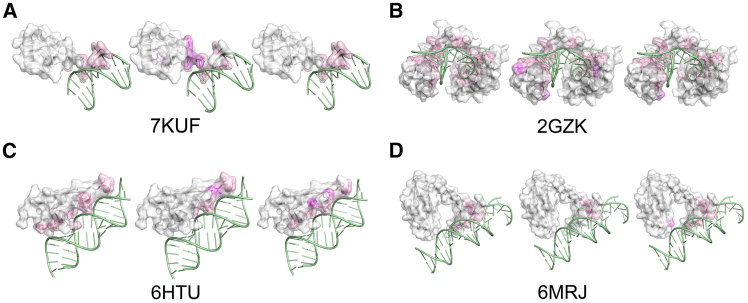
Case-study of PNABPred Case studies illustrating PNABPred’s accuracy on diverse protein-nucleic acid complexes, where left represents the experimental result, and middle and right represent the results predicted by CLAPE and PNABPred, respectively. True positive binding sites (pink surface) and false positive binding sites (peach surface) are mapped onto protein structures for: (A) the bacterial transcription factor WhiB7 (PDB: 7KUF), (B) the chimeric HMGB protein with disordered binding regions (PDB: 2GZK), (C) the human Staufen1 dsRNA-binding domain (PDB: 6HTU), and (D) the nickel-responsive regulator NikR from *Helicobacter pylori* (PDB: 6MRJ).

To evaluate PNABPred’s capacity to classify diverse NABPs and identify binding sites, we analyzed a representative set of protein-nucleic acid complexes (Figure 4; Figure S5). For DBPs, our selection included the classic bacteriophage 434 Cro protein (PDB: 3CRO), a key regulator in the phage lytic-lysogenic switch, as well as a chimeric high-mobility group box (HMGB) protein (PDB: 2GZK), with IDRs known to participate in DNA binding. We also selected a group of prokaryotic transcriptional regulators that function through distinct mechanisms, including TetR family regulators from *Mycobacterium tuberculosis* (PDB: 6C31) and the archaeon *Sulfolobus acidocaldarius* (PDB: 6EN8), as well as the nickel-responsive regulator, NikR from *Helicobacter pylori* (PDB: 6MRJ). We also included the bacterial transcription activation subcomplex containing the regulator WhiB7 bound to DNA (PDB: 7KUF). For RBPs, we examined the RNA-binding domain of the yeast transcription termination factor Nrd1 (PDB: 5O1Y) and the double-stranded RNA-binding domain from human Staufen1 (PDB: 6HTU).

PNABPred demonstrated high and biologically relevant accuracy across these varied systems. Analysis of 3CRO showed that PNABPred could correctly identify R and K DNA-interacting residues that form electrostatic interactions with the DNA backbone. Examining the more flexible chimeric HMGB protein 2GZK, we found that PNABPred also identified the majority of true binding sites, while most false positives were located in regions spatially adjacent to the true interaction interface. This ability to learn broader, functionally relevant regions supported its accuracy across heterogeneous protein datasets. Similarly, PNABPred successfully pinpointed conserved DNA-binding domains of other prokaryotic transcriptional regulators (i.e., 6C31, 6EN8, and 6MRJ). The model’s performance was particularly noteworthy in correctly identifying all DNA-binding sites on the WhiB7 regulator, 7KUF. Among representative RBPs, PNABPred accurately predicted binding sites in both yeast Nrd1 (5O1Y) and human hStau1 (6HTU) and correctly detected enrichment of residues such as R and H. These case studies collectively validate the high precision of PNABPred across various organisms and binding modes.

We also analyzed a set of 14 representative proteins, comprising specific transcription factors (TFs), specific RBPs, and non-specific DBPs (e.g., Histones). The model exhibits distinct predictive patterns consistent with the underlying biophysics (Figures S6 and S7). For specific binders, particularly RBPs, predictions are highly localized (average interface coverage ∼10%) and rely less on pure electrostatics (Lys/Arg ratio ∼25%), indicating recognition is driven by diverse residue types fitting a specific pocket. In contrast, for non-specific binders like histones, PNABPred predicts broader binding surfaces (coverage up to ∼26%) that are enriched in basic residues (Lys/Arg ratio >40%), consistent with an affinity mechanism driven largely by electrostatic interactions (generic electrostatic patches) with the nucleic acid backbone. These findings shows that PNABPred effectively captures functional distinctions beyond simple electrostatic matching.

## Discussion

In this study, we introduce PNABPred, a multi-modal framework relying on protein sequence alone to accurately classify NABPs and predict their specific binding residues. Evaluation on multiple benchmark datasets indicated that PNABPred exhibits stronger performance than existing state-of-the-art sequence-based methods and is competitive with structure-based approaches. These findings also suggest that multi-modal sequence representations in our model implicitly encode spatial proximity and chemical environment information necessary for accurate prediction of nucleic acid binding activity. This advantage suggests that PNABPred may be a particularly promising tool for analyzing IDRs, which are poorly handled by current structure-based predictors due to their lack of stable tertiary structure.^53^ Overcoming this obstacle, PNABPred allows proteome-wide analysis of nucleic acid binding, thus providing a means for large-scale functional annotation. By integrating biophysical and evolutionary features with the pLM, PNABPred achieves the highest in accuracy and generalizability for sequence-based models and provides a framework for building robust predictive models that utilize sequence alone to predict other protein functions.

Although pLM can serve as powerful foundations for protein modeling, our ablation studies conclusively demonstrate that supplementation with manually curated biological knowledge can further enhance predictive performance. Through the multi-modal representation learning approach proposed here, we could integrate a variety of protein- and residue-level features to improve pLM accuracy and generalizability. Although we applied current understanding of specific residue properties involved in nucleic acid binding to enhance pLM representation, knowledge of motif-level interactions might further improve prediction accuracy or model capabilities. For example, human-defined regular expressions with known NABPs can be used to detect DNA/RNA binding motifs in sequencing data.^54^ Incorporating features identified through such methods with our binding site prediction strategy may further improve accuracy. Similarly, including Gene Ontology (GO) terms^55^^,^^56^ and other functional knowledge might also increase prediction accuracy. This framework supports the integration of diverse prior information to enhance the performance of pLMs.

### Limitations of the study

We acknowledge, however, that PNABPred focuses solely on the protein interface. While it accurately predicts binding propensity and residue sites, it does not infer the specific nucleic acid sequence motifs (e.g., k-mers) that the protein recognizes, as the model does not take nucleic acid sequences as input. Because predictions rely solely on protein sequence, they reflect the intrinsic binding potential of residues defined by physicochemical and evolutionary properties, rather than capturing dynamic, ligand-specific induced fit. This characteristic renders the model particularly valuable for identifying potential binding pockets without requiring prior knowledge of the specific ligand. Further, as with many computational models, its predictions are based on curated data and do not explicitly account for the *in vivo* cellular context, such as the influence of post-translational modifications. Following this logical progression, in future work, the PNABPred framework could be extended to predict protein specificity for different nucleic acid substrates, potentially by employing a dual-encoder architecture that processes both protein and nucleic acid sequences. The resulting framework could be modified to predict nucleic acid motifs, further enabling prediction of DNA specificity relevant to the discovery of new TFs and biological pathways.^57^ Alternatively, RNA specificity predictions could facilitate the design of RNA aptamers for therapeutic or biotechnology applications.^58^

Moreover, many TFs (e.g., bHLH and bZIP families) function as dimers or multimers. Although PNABPred operates on single sequences, it effectively captures the intrinsic binding potential of these monomers (Figures S6 and S7). However, the performance on obligate dimers suggests that, where the continuous binding interface is fully stabilized only upon oligomerization, the model may exhibit reduced sensitivity at the interface boundaries compared with structurally self-contained monomers. Future architectural enhancements could address this by employing multi-chain input strategies, such as concatenating subunit sequences, to better capture features strictly dependent on quaternary assembly. It is also noteworthy that our framework could be adapted to other protein-ligand interaction prediction problems beyond NABPs, such as identifying protein-small molecule binding sites, protein-peptide binding sites, protein-protein interaction sites, enzyme catalytic residues, and other functional sites. Such protein-ligand prediction problems, common in data mining, functional genomics, and drug development research, might be feasible through switching only specific datasets without altering the model architecture, presenting a scalable tool for annotation and screening of sequence data.

## Resource availability

### Lead contact

Further information and requests for resources and reagents should be directed to and will be fulfilled by the lead contact, Boxue Tian (boxuetian@mail.tsinghua.edu.cn).

### Materials availability

This study did not generate new unique reagents.

### Data and code availability

• Data: The datasets used in this study are available at https://doi.org/10.5281/zenodo.19572111
• Code: The source code for PNABPred has been deposited at https://doi.org/10.5281/zenodo.19572111
• Any other items: Any additional information required to reanalyze https://doi.org/10.5281/zenodo.19572111

## Acknowledgments

This work was supported by the 10.13039/501100004147Tsinghua University Initiative Scientific Research Program (no. 20251080016) and the Tsinghua-10.13039/501100007937Peking University 10.13039/501100011620Center for Life Sciences (no. 20111770319).

## Author contributions

Z.S. designed the framework and performed most of the experiments, including data preprocessing, model training, and manuscript writing; X.Z. helped with data preprocessing; B.T. directed the study and helped the authors write the manuscript.

## Declaration of interests

The authors declare no competing interests.

## STAR★Methods

### Key resources table

**Table:** 

REAGENT or RESOURCE	SOURCE	IDENTIFIER
**Deposited data**		

PNABPRED SOURCE CODE AND DATASETS	This paper	https://doi.org/10.5281/zenodo.19572111
BIOLIP DATABASE (USED FOR NEW TEST SETS)	Yang et al.^50^	https://zhanggroup.org/BioLiP
UNISWISS DATASET (DBP TRAINING/TESTING)	Hu et al.^35^; Zeng et al.^36^	N/A
RBPPRED DATASET (RBP TRAINING/TESTING)	Zhang & Liu^37^	http://rnabinding.com/RBPPred.html
SEQ-RBPPRED DATASET (RBP TRAINING/TESTING)	Yan et al.^38^	https://github.com/yaoyao-11/Seq-RBPPred
TEST_46, TEST_129, TEST_117 (GRAPHBIND DATASETS)	Xia et al.^19^	http://www.csbio.sjtu.edu.cn/bioinf/GraphBind
PDB (PROTEIN DATABANK) STRUCTURES	RCSB PDB	https://www.rcsb.org

**Software and algorithms**		

PNABPRED	This paper	https://doi.org/10.5281/zenodo.19572111
ESM-2 (PROTEIN LANGUAGE MODEL)	Lin et al.^20^	https://github.com/facebookresearch/esm
LORA (LOW-RANK ADAPTATION) CODE	Hu et al.^39^	https://github.com/microsoft/LoRA

### Method details

#### Datasets

To directly compare the performance of PNABPred with prior works, we utilized multiple publicly available benchmark datasets (Tables S1 and S2) for both protein classification and residue binding site prediction tasks. For protein classification, we used the UniSwiss^35^^,^^38^ dataset for DNA-binding proteins (DBPs) and the RBPPred^41^ and Seq-RBPPred^36^ dataset for RNA-binding proteins (RBPs). For residue binding site prediction, we used standard datasets previously employed in GraphBind and DBPred, including Test_46 and Test_129 for DNA-binding sites, and Test_117 for RNA-binding sites. We used Test_181 to evaluate cross-dataset generalization as an additional independent test set. To assess the performance on contemporary proteins, absent in established benchmarks, two new test datasets were constructed using data from the BioLiP database, with an update cutoff of January 2025. Following the data processing protocol of GraphBind, all protein sequences in the above-mentioned sets were filtered via CD-HIT to ensure a maximum of 30% sequence identity against our training data. We also removed overlaps among the different test sets and standard benchmarks guaranteeing independence and rigorous generalization testing. For all datasets, binding sites were defined as residues containing an atom within a distance of 0.5 Å plus the sum of the van der Waals radii of the two closest atoms (one from the residue and one from the nucleic acid molecule). All datasets underwent uniform preprocessing to ensure data quality and to facilitate a reliable and fair performance evaluation.

#### Multi-modal feature representation

PNABPred processes each protein sequence using three embedding methods to capture distinct and complementary features.

ESM-2 Embeddings: To generate contextual protein representations, we used the pre-trained protein language model ESM-2, a 33-layer Transformer with 650 million parameters. For each input protein sequence, we extracted the per-residue embeddings from the final (33rd) layer of the model. These embeddings capture high-level semantic information, resulting in a 1280-dimensional vector for each amino acid.

Atchley Factors Embeddings: To incorporate fundamental biophysical information, we used Atchley factors, which reduced approximately 500 different amino acid attributes into a 5-dimensional vector that represents five key properties: (I) polarity, (II) secondary structure propensity, (III) molecular volume, (IV) codon diversity, and (V) electrostatic charge. For each amino acid in a sequence, its 5-dimensional Atchley factors vector was projected into a 512-dimensional embedding space using a linear layer, followed by a ReLU activation function and Layer Normalization.

BLOSUM50 Matrix Embeddings: To provide explicit evolution-inspired priors, we utilized the BLOSUM50 substitution matrix, which contained log-odds scores for the substitution of one amino acid for another, derived from alignments of conserved protein blocks with less than 50% identity, making it suitable for capturing relationships between divergent sequences:

(Equation 1) $$\text{score}\left(A,B\right)=\log_{2}\left(\frac{f\left(A\to B\right)}{f\left(A\right)\times f\left(B\right)}\right)$$

where score(*A*,*B*)is the substitution score for amino acids *A* and *B*, *f*(*A*→*B*) is the observed substitution probability, and *f*(*A*) and *f*(*B*) are background frequencies. For each residue, we created a 20-dimensional vector of its substitution scores against all standard amino acids. This vector was then mapped to a 512-dimensional space using a linear layer with ReLU and LayerNorm.

Positional Encoding and Intra-Modal Refinement: Positional encodings were added to the Atchley and BLOSUM embeddings to provide the model with information about residue order. To further enhance these two feature streams, each embedding was independently processed by an 8-head self-attention layer before the main fusion stage. This intra-modal refinement step allowed the model to learn context-dependent representations within each modality, enriching the features by capturing not just the inherent properties of residues but also their sequence-specific significance.

#### The PNABPred framework architecture

The PNABPred framework is composed of two main stages: a multi-modal feature embedding module that generates rich representations for each amino acid, and an attention-based backbone network that fuses these features and performs prediction.

The three feature streams are concatenated along the feature dimension for each residue, combining the 1280-dimensional contextual embedding directly from ESM-2, the 512-dimensional context-refined Atchley Factors embedding, and the 512-dimensional context-refined BLOSUM50 Matrix embedding into a single 2304-dimensional feature vector per residue, which serves as the input for the backbone network.

##### Backbone network

The backbone of PNABPred is a multi-layer Transformer encoder. This architecture is built upon the self-attention mechanism, which allows the model to weigh the importance of all other residues in the sequence when producing the representation for a given residue. For each input, Query (Q), Key (K), and Value (V) vectors are generated. Attention scores are computed via the scaled dot-product of the Query with all Keys, followed by normalization to produce attention weights. These weights are subsequently applied to compute a weighted aggregation of Value vectors, enabling the model to capture complex long-range dependencies. The architecture consists of multiple stacked layers, each containing an 8-head self-attention module and a position-wise feedforward network. Layer Normalization and Dropout are applied within each layer to stabilize training and prevent overfitting.

##### Classification head

The final hidden state representation for each residue from the Transformer backbone was passed to a classification head. This head, a Multi-Layer Perceptron (MLP) consisting of several linear layers with ReLU activations and Dropout. The MLP outputs a two-dimensional logit vector for each residue, corresponding to the scores for the non-binding and binding classes.

#### Parameter-efficient fine-tuning and model training

Low-Rank Adaptation (LoRA) for Fine-Tuning: To efficiently adapt the large ESM-2 model to our specific tasks, we employed Low-Rank Adaptation (LoRA),^59^ a parameter-efficient fine-tuning (PEFT) technique. Instead of fine-tuning all of the model’s weights, LoRA freezes the pre-trained weights and injects smaller, trainable rank-decomposition matrices into specific layers of the Transformer architecture. The update to a weight matrix *W*is reparameterized as a low-rank product

(Equation 2) W+ΔW=W+BA

where *B* and *A* are the newly selected trainable matrices and the rank *r* is much smaller than the original dimensions. Δ*W* can be approximated by the product of two much smaller, low-rank matrices:

(Equation 3) ΔW≈BA

where *W*, Δ*W*∈*R*^*d*×*k*^,*B*∈*R*^*d*×*r*^and *A*∈*R*^*r*×*k*^, with the rank *r*≪*d*,*k*. This approach dramatically reduces the number of trainable parameters by orders of magnitude, making fine-tuning computationally feasible while maintaining performance compared to full fine-tuning. We applied LoRA to the *Query*(*Q*) and *Value*(*V*)projection matrices within the multi-head self-attention modules of the ESM-2 Transformer layers. This strategy not only improved efficiency but also acted as a regularizer, preventing the model from overfitting to the training data and avoiding catastrophic forgetting of the general biological knowledge encoded in the pre-trained weights.

Training Procedure: The model was trained end-to-end using the cross-entropy loss function with the AdamW optimizer. The hyperparameter settings, including the learning rate, batch size, number of epochs, and LoRA specifications, are listed in Table S10. The model training was completed on an NVIDIA A100 GPU. To ensure reproducibility, we repeated the training process 10 times. For each run, we used a different random seed for weight initialization and data shuffling.

### Quantification and statistical analysis

#### Evaluation and statistical analysis

Evaluation Metrics: Model performance was assessed using a comprehensive set of metrics: Precision (Pre), F1 score, Matthews correlation coefficient (MCC), and area under the receiver operating characteristic curve (AUROC), providing a comprehensive assessment:

(Equation 4) $$Precision=\frac{TP}{TP+FP}$$

(Equation 5) $$Recall=\frac{TP}{TP+FN}$$

(Equation 6) $$F1=\frac{2\cdot Rec\cdot Pre}{Rec+Pre}$$

(Equation 7) $$MCC=\frac{TP\cdot TN-FN\cdot FP}{\sqrt{\left(TP+FN\right)\left(TP+FP\right)\left(TN+FN\right)\left(TN+FP\right)}}$$

Here, TP, FP, TN, and FN denote true positives, false positives, true negatives, and false negatives, respectively. Precision measures the proportion of true positives among predicted positives, F1 balances precision and recall, MCC provides a comprehensive performance metric, and AUROC assesses overall discriminative ability independent of classification thresholds. These metrics were chosen for consistency and direct comparability with the performance reported in prior studies on the same benchmark datasets.

##### Statistical analysis

To ensure statistical validity and reproducibility, we adopted a testing protocol based on independent replication. The statistical sample consisted of performance metrics derived from 10 independent training runs. To enable a fair distribution-based comparison, we trained both PNABPred and the second-best performing baseline model (e.g., CLAPE) 10 times using identical data splits and evaluation protocols. In each run, a different random seed was used for weight initialization and data shuffling to capture model variance. We employed the non-parametric Mann-Whitney U test (two-sided) to assess whether the performance distribution of PNABPred was significantly different from that of the reproduced baseline. We also applied the Benjamini-Hochberg (BH) procedure. All reported *p*-values were adjusted, with a significance threshold set at 0.05.
