# Supplementary material for: Predicting protein-nucleic acid interactions via protein language models with biophysical and evolutionary priors
Source: iScience. 2026 Apr 17;29(5):115795. doi: 10.1016/j.isci.2026.115795 (PMC13146549; doi:10.1016/j.isci.2026.115795)
Supplement: Document S1. Figures S1–S7 and Tables S1–S10 [file mmc1.pdf]

iScience, Volume *29*

Supplemental information

Predicting protein-nucleic acid interactions via protein language models with biophysical and evolutionary priors

Zidong Su, Xiaochun Zhang, and Boxue Tian

Contents

Figure S1: Performance comparison of different backbone architectures

Figure S2: t-SNE visualization of original ESM-2 embeddings

Figure S3: Comparison of amino acid frequencies

Figure S4: Heatmap of amino acid binding ratios

Figure S5: Additional case studies of PNABPred performance

Figure S6: Visualization of predicted binding sites on multimeric and normal/non-specific binding proteins

Figure S7: Analysis of binding site distributions

Table S1: Overview of benchmark datasets for protein-level classification

Table S2: Overview of benchmark datasets for residue-level binding site prediction

Table S3: Overview of newly-curated and independent test datasets

Table S4: Test performance on generalization assessment datasets

Table S5: Statistical Significance Analysis (p-values) for protein-level classification benchmark datasets with Mann-Whitney U Test and BH Correction

Table S6: Statistical Significance Analysis (p-values) for residue-level binding site prediction benchmark datasets with Mann-Whitney U Test and BH Correction

Table S7: Ablation studies for protein-level classification

Table S8: Ablation studies for residue-level binding site prediction

Table S9: Performance comparison of different backbone architectures

Table S10. Hyperparameters and training configurations of the PNABPred framework

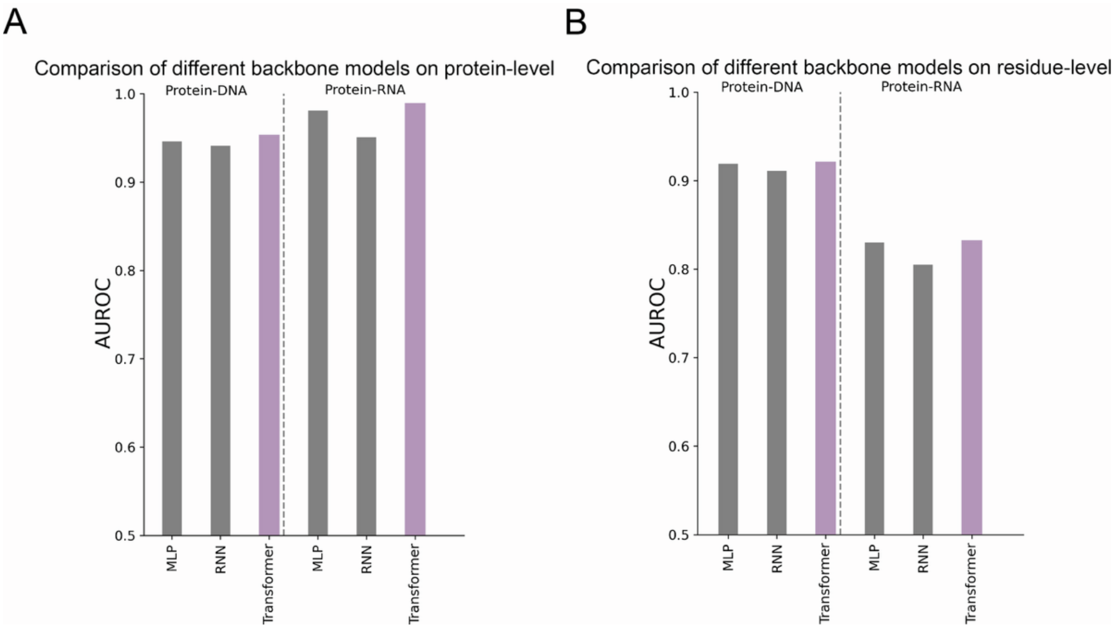

Figure S1. Performance comparison of different backbone architectures. Performance of the Transformer backbone compared to Multi-Layer Perceptron (MLP) and Recurrent Neural Network (RNN) architectures on (A) protein-level classification and (B) residue-level binding site prediction tasks.

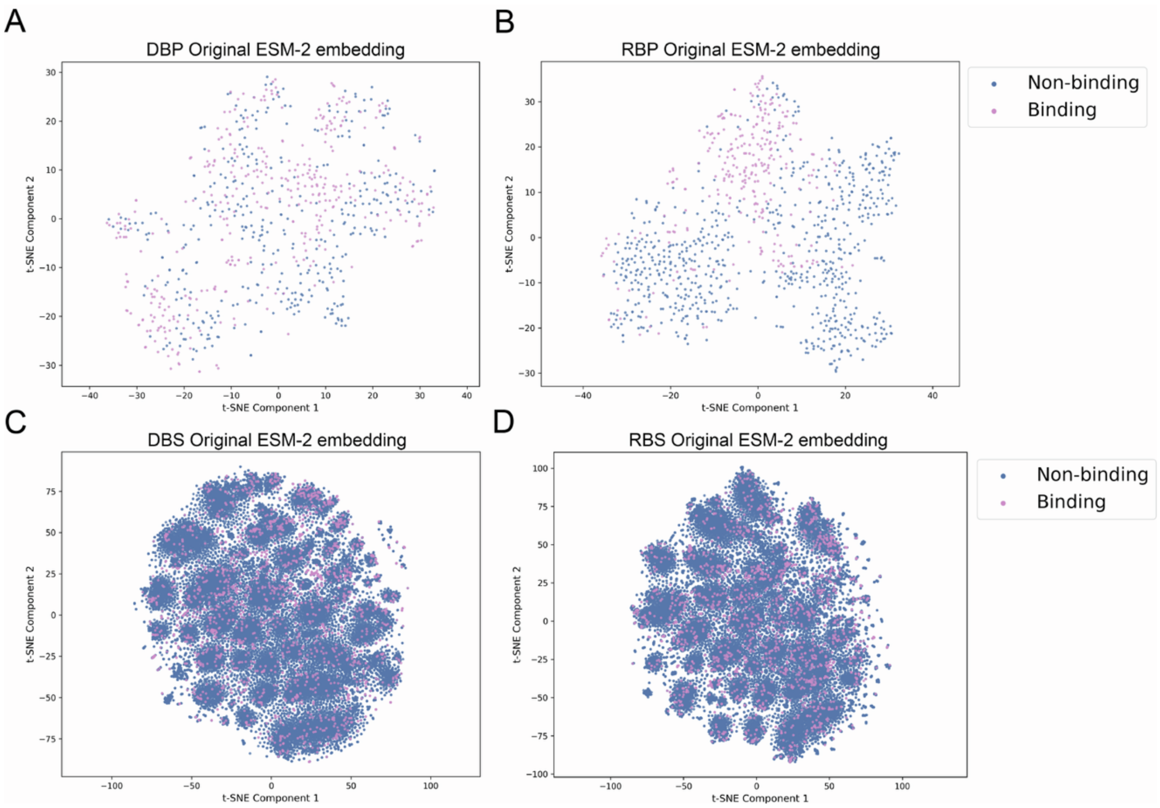

Figure S2. t-SNE visualization of original ESM-2 embeddings. (A) Visualization for the DNA-binding protein (DBP) classification task. (B) Visualization for the RNA-binding protein (RBP) classification task. (C) Visualization for the DNA-binding site (DBS) prediction task. (D) Visualization for the RNA-binding site (RBS) prediction task. Binding residues/proteins are shown in pink and non-binding in blue.

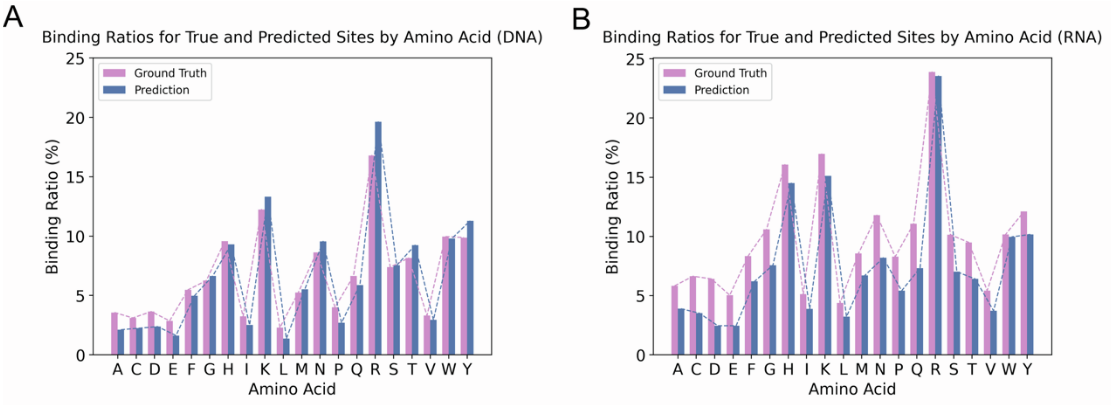

Figure S3. Comparison of amino acid frequencies. (A, B) Comparison of amino acid frequencies at predicted (blue bars) versus ground-truth (pink bars) binding sites for (A) DNA and (B) RNA. The model's predictions closely replicate the true distributions, capturing known biophysical preferences like the enrichment of positively charged (R, K) and aromatic (W) residues.

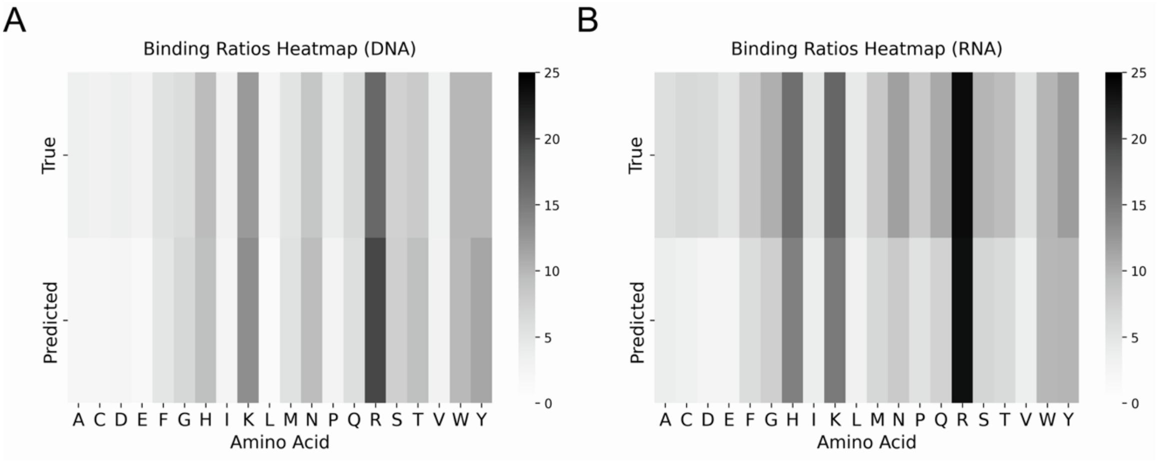

Figure S4. Heatmap of amino acid binding ratios. Heatmaps comparing the frequency distribution of amino acids at true binding sites versus PNABPred-predicted binding sites. The color intensity corresponds to the binding ratio percentage, with darker shades indicating higher frequency. (A) Comparison for DNA-binding sites. (B) Comparison for RNA-binding sites.

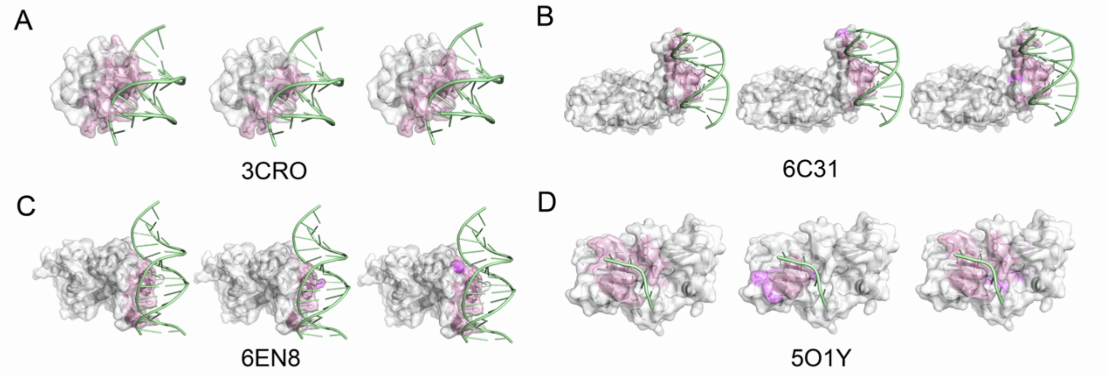

Figure S5. Additional case studies of PNABPred performance. Additional case studies illustrating PNABPred's accuracy on diverse protein-nucleic acid complexes, where left represents the experimental result, middle and right represent the results predicted by CLAPE and PNABPred, respectively. True positive binding sites (pink surface) and False positive binding sites (peach surface) are mapped onto protein structures for: (A) Bacteriophage 434 Cro protein (PDB: 3CRO). (B) TetR family transcriptional regulator from Mycobacterium tuberculosis (PDB: 6C31). (C) TetR family regulator from Sulfolobus acidocaldarius (PDB: 6EN8). (D) RNA-binding domain of yeast transcription termination factor Nrd1 (PDB: 5O1Y).

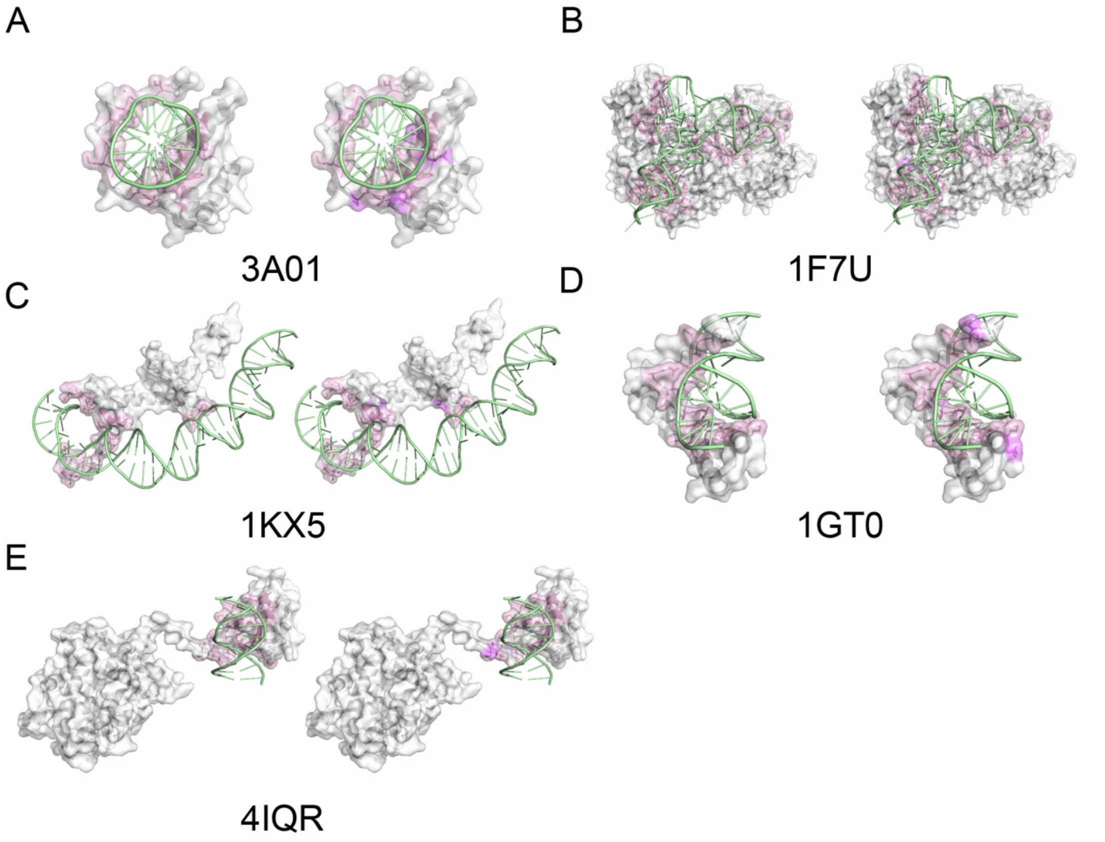

Figure S6. Visualization of predicted binding sites on multimeric and normal /non-specific binding proteins. Comparison of experimentally determined binding sites (left) and PNABPred-predicted binding sites (right) mapped onto 3D structures. Nucleic acid ligands are shown in green, and binding sites are rendered as pink surfaces. (A) Basic Helix-Loop-Helix (bHLH) transcription factor dimer (PDB: 3A01). (B) Specific RNA-binding protein U1A (PDB: 1F7U). (C) Nucleosome core particle (Histones) representing non-specific DNA binding (PDB: 1KX5). (D) Basic Leucine Zipper (bZIP) transcription factor dimer (PDB: 1GT0). (E) Nuclear Receptor DNA-binding domain dimer (PDB: 4IQR).

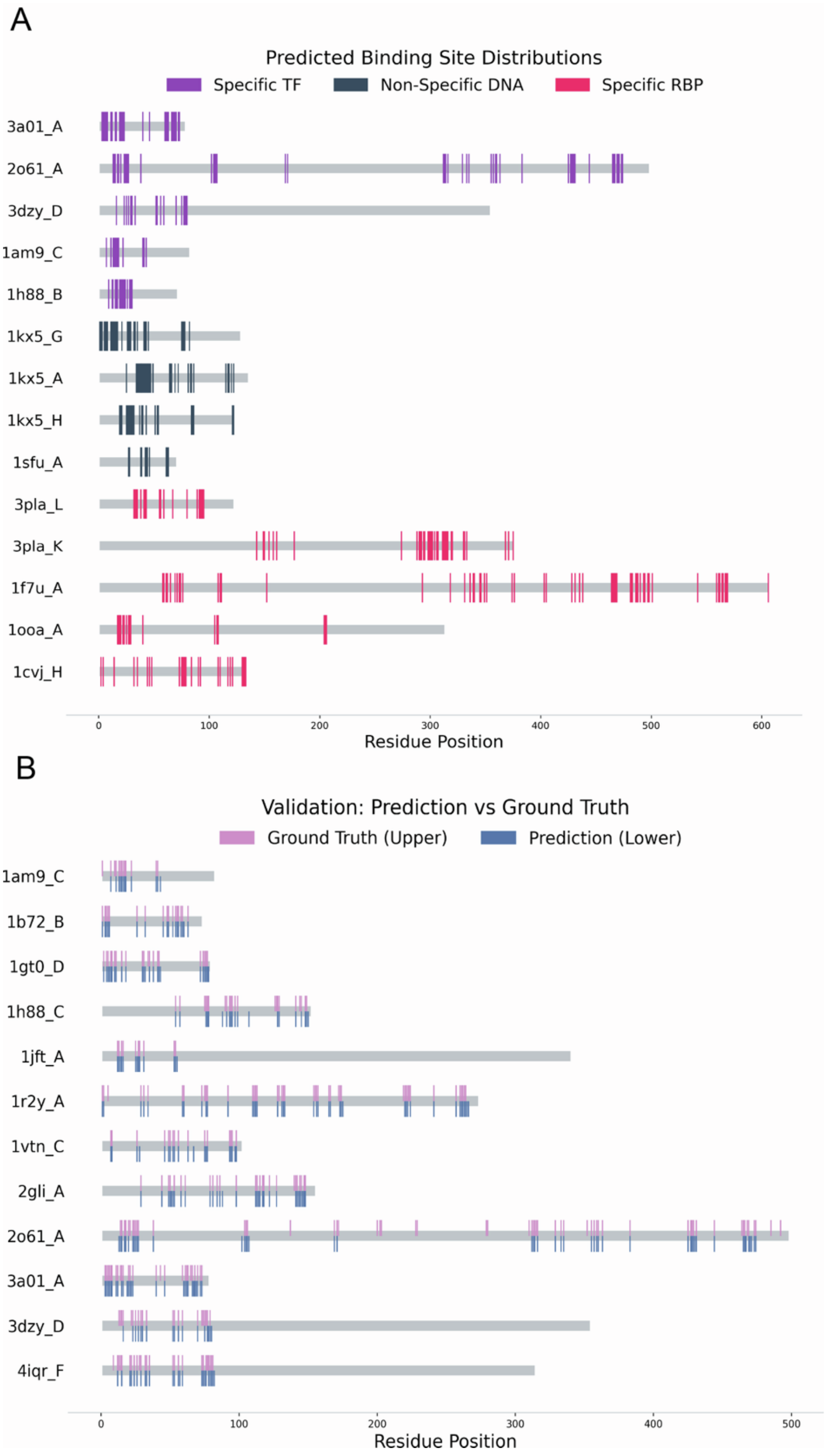

Figure S7. Analysis of binding site distributions. (A) Distribution of predicted binding residues along protein sequences for three functional categories: Specific Transcription Factors (purple), Non-Specific DNA-binding proteins (dark blue), and Specific RNA-Binding Proteins (red). (B) Residue-level comparison of Ground Truth (pink, upper row) versus PNABPred predictions (blue, lower row) for a curated set of 12 dimeric/multimeric transcription factors.

Table S1. Overview of benchmark datasets for protein-level classification

Datasets	Protein-DNA Train	Protein-DNA Test	Protein-RNA Train	Protein-RNA Test
Binding Protein	4881	376	2780	275
Non- Binding Protein	4881	376	7093	695
% of Binding Protein	50.00	50.00	28.16	28.35

Table S2. Overview of benchmark datasets for residue-level binding site prediction

Datasets	Train_646	Test_46	Train_573	Test_129	Train_495	Test_117
Binding residues	15636	965	14479	2240	14609	2031
Non-Binding residues	298503	9911	145404	35275	122290	35314
% of Binding residues	4.98	8.87	9.06	5.97	10.67	5.44

Table S3. Overview of newly-curated and independent test datasets

Datasets	TrainDNA_2025	TestDNA_2025	TrainRNA_2025	TestRNA_2025	Test_181
Binding residues	22364	2393	55341	5883	3208
Non-Binding residues	436018	49183	652233	80400	72050
% of binding residues	4.88	4.64	7.82	6.82	4.26

Table S4. Test performance on generalization assessment datasets

	Datasets	Models	Pre	F1	MCC	AUROC
Protein–DNA	TestDNA_2025	CLAPE-DB	0.356	0.342	0.309	0.808
		PNABPred	0.547±0.004	0.470±0.005	0.451±0.003	0.922±0.001
	Test_181	DNAPred	0.223	0.267	0.233	0.802
		SVMnuc	0.242	0.263	0.229	0.803
		NCBRPred	0.241	0.250	0.215	0.771
		CLAPE-DB(Train_646)	0.212	0.280	0.252	0.785
		CLAPE-DB(Train_573)	0.288	0.341	0.287	0.824
		PNABPred (Train_646)	0.438±0.002	0.371±0.009	0.344±0.003	0.892±0.001
		PNABPred (Train_573)	0.356±0.012	0.384±0.001	0.357±0.001	0.894±0.001
Protein–RNA	TestRNA_2025	CLAPE-DB	0.361	0.279	0.268	0.768
		PNABPred	0.583±0.008	0.413±0.008	0.400±0.005	0.880±0.001

Table S5. Statistical Significance Analysis (p-values) for protein-level classification benchmark datasets with Mann-Whitney U Test and BH Correction

Comparison	DBP	RBP
PNABPred vs LBi-DBP	0.012	-
PNABPred vs Seq-RBPPred	-	0.012

Table S6. Statistical Significance Analysis (p-values) for residue-level binding site prediction benchmark datasets with Mann-Whitney U Test and BH Correction

Comparison	Test_46	Test_129	Test_117	TestDNA_2025	TestRNA_2025
PNABPred vs CLAPE	0.012	0.012	0.012	0.012	0.012

Table S7. Ablation studies for protein-level classification

Datasets	Models	MCC	AUROC
DBP	Without attention module	0.811	0.951
	Without pLM module	0.539	0.798
	Without Biophysical module	0.793	0.949
	Without Evolutionary module	0.792	0.947
	pLM Only	0.785	0.951
	Original Model	0.825	0.959
RBP	Without attention module	0.885	0.988
	Without pLM module	0.582	0.887
	Without Biophysical module	0.884	0.895
	Without Evolutionary module	0.847	0.982
	pLM Only	0.876	0.988
	Original Model	0.889	0.990

Table S8. Ablation studies for residue-level binding site prediction

Datasets	Models	MCC	AUROC
DBS(Test_129)	Without attention module	0.461	0.920
	Without pLM module	0.124	0.709
	Without Biophysical module	0.460	0.917
	Without Evolutionary module	0.457	0.918
	pLM Only	0.459	0.920
	Original Model	0.468	0.922
RBS(Test_117)	Without attention module	0.240	0.830
	Without pLM module	0.072	0.681
	Without Biophysical module	0.239	0.830
	Without Evolutionary module	0.221	0.823
	pLM Only	0.241	0.828
	Original Model	0.256	0.833

Table S9. Performance comparison of different backbone architectures

Datasets	Models	MCC	AUROC
DBP	MLP	0.787	0.946
	RNN	0.763	0.941
	Transformer	0.810	0.953
RBP	MLP	0.875	0.981
	RNN	0.878	0.951
	Transformer	0.889	0.989
DBS(Test_129)	MLP	0.457	0.919
	RNN	0.434	0.911
	Transformer	0.462	0.921
RBS(Test_117)	MLP	0.249	0.830
	RNN	0.220	0.805
	Transformer	0.256	0.832

Table S10. Hyperparameters and training configurations of the PNABPred framework

Datasets	Models	MCC	AUROC
Training	Batch Size	Number of samples processed per iteration	[16, 32]
	Learning Rate	Initial learning rate for AdamW optimizer	[1e-4, 5e-5]
	Epochs	Maximum number of training epochs	[50, 100, 200]
	Weight Decay	Coefficient for L2 regularization	[1e-4, 1e-5]
	Dropout	Dropout probability in the classification head	[0.1, 0.2]
LoRA (PEFT)	LoRA Rank (r)	Rank of the low-rank adaptation matrices	[4, 8]
	LoRA Alpha	Scaling factor for LoRA weights	[8, 16]
	Target Modules	Transformer layers where LoRA is applied	Query, Value
Backbone	ESM-2 Model	Pre-trained protein language model version	esm2_t33_650M_UR50D
	Max Sequence Length	Maximum input length	1024

Hyperparameters were tuned on the validation set within the specified ranges for each dataset
